# Development and Characterization of a Novel Soil Amendment Based on Biomass Fly Ash Encapsulated in Calcium Alginate Microspheres

**DOI:** 10.3390/ijms23179984

**Published:** 2022-09-01

**Authors:** Marko Vinceković, Suzana Šegota, Slaven Jurić, Maria Harja, Gabrijel Ondrasek

**Affiliations:** 1 Department of Chemistry, The University of Zagreb Faculty of Agriculture, Svetošimunska c. 25, 10000 Zagreb, Croatia; 2 Ruđer Bošković Institute, Laboratory for Biocolloids and Surface Chemistry, Bijenička c. 54, 10000 Zagreb, Croatia; 3 Department of Chemical Engineering, “Gheorghe Asachi” Technical University of Ias, Bulevardul Profesor Dimitrie Mangeron 67, 700050 Iași, Romania; 4 Department of Soil Amelioration, The University of Zagreb Faculty of Agriculture, Svetošimunska c. 25, 10000 Zagreb, Croatia

**Keywords:** biomass fly ash, encapsulation, calcium alginate, soil amendment, recycling of natural resources

## Abstract

Biomass fly ash (BFA) from a biomass cogeneration plant was encapsulated into calcium alginate microspheres (ALG/Ca) and characterized. An FTIR analysis indicated that BFA loading weakened molecular interactions between ALG/Ca constituents (mainly hydrogen bonding and electrostatic interactions), thus changing the crosslinking density. SEM and AFM analyses revealed a wrinkled and rough surface with elongated and distorted granules. The in vitro release of BFA’s main components (K, Ca, and Mg) was controlled by diffusion through the gel-like matrix, but the kinetics and released amounts differed significantly. The smaller released amounts and slower release rates of Ca and Mg compared to K resulted from the differences in the solubility of their minerals as well as from the interactions of divalent cations with alginate chains. The physicochemical properties of the novel microsphere formulation reveal significant potential for the prolonged delivery of nutrients to crops in a safe manner.

## 1. Introduction

Large amounts of biomass fly ash (BFA) generated by plant biomass combustion for electricity and thermal energy generation represent a global management problem. It was estimated that 10 million tons of BFA are produced annually in the world (data from 2017) [1]. Recycling and reusing biomass fly ash as other materials is an option for reducing the amount to be disposed. BFA is a valuable source of inorganic material, and it is already in use for various industrial purposes (the cement industry, construction, embankments, etc.) as well as in agriculture and forestry [2,3]. The advantages of applying biomass fly ash in agriculture are the improvement in the physical, chemical, and biological properties of degraded soils and the delivery of micro- and macronutrients (K, Na, Zn, Ca, Mg, Fe, etc.) to plants, which increases crop productivity [4]. Due to the high content of plant nutrients, BFA meets the minimum requirements for mineral fertilizers despite a small proportion of nitrogen [5]. The application of biomass fly ash has stimulated, for example, an increase in the growth and yield of barley [6], maize [7], etc. Besides increasing the yields of many crops, biomass fly ash often contains trace concentrations of heavy metals (Cu, Cd, Ni, Pb, Hg, As, etc.), leading to possible toxic accumulation in crops, and this should be properly monitored [8,9]. Biomass ashes exhibit significant variations in composition, properties, and characteristics depending on plant species, the origin of the plant, the process parameters during incineration, and the storage conditions of the combustion residues [10,11]. Due to a significant amount of minerals and plant nutrients as well as a reactive surface capable of interacting with potentially toxic elements [12], BFA can be used as an economical fertilizer or soil amendment. The recycling of BFA in agroforestry ecosystems is one example of the sustainable use of natural resources [13]. The advantages are fast growth and an increase in biomass and wood quality.

Very fine biomass fly ash particles create problems when applied in the field or on larger surfaces, so it is necessary to use special and expensive applicators for powder materials to protect against inhalation, wind drift, and uneven application on the ground, which can lead to the burning of plant surfaces, etc. A better alternative to applicators is fly ash granules, which are usually used as a soil conditioner material [14], but the effect of fertilization is reduced [15]. Moreover, one of the problems with the use of fly ash is that, despite the positive impact on soil quality and plant growth and a significant increase in yield, it is important to apply it in a concentration that is beneficial because larger amounts can be harmful due to a possible sudden increase in soil alkalinity or nutrient leachability [16]. An elegant solution would be to use a controlled-release method such as encapsulation technology, which inserts the substance of interest into the carrier matrix, delivering it to the selected site at the appropriate concentrations and prolonging the time that plants must absorb the nutrients. Encapsulation in biopolymer matrices has already been recognized as an effective method for the prolonged release of micro- and macronutrients as well as microorganisms for plant nutrition and protection [17,18]. Despite many methodologies for the encapsulation of chemical or biological agents, there are no data in the literature on BFA encapsulation in biopolymer microspheres for slow plant nutrient release to the soil.

The main goal of this research is to use encapsulation as a more acceptable approach for handling BFA during storage, transport, and application in agriculture. In this work, BFA was loaded in biopolymeric microspheres prepared by the ionic gelation of alginate with calcium. The preparation of a calcium alginate microsphere is simple, and its components are easily accessible. It is merely a slow-release system (controls the release of the encapsulated ingredient over time) and can be tailored according to the ingredient being encapsulated as well as the application requirements [18]. In addition to the possibility of delivering encapsulated bioactive substances or microorganisms to the plants, biopolymer alginate also plays the role of a soil amendment, modifying the condition of the soil by encouraging soil water retention to elicit a defensive response that results in protection against pathogens or insect damage [19]. The prepared microsphere formulation has the potential to deliver nutrients to plants over an extended period of time and represents the safe recycling of nutrients removed from the environment.

## 2. Results and Discussion

### 2.1. Molecular Interaction between Constituents in Microsphere Formulation

**Figure 1:**
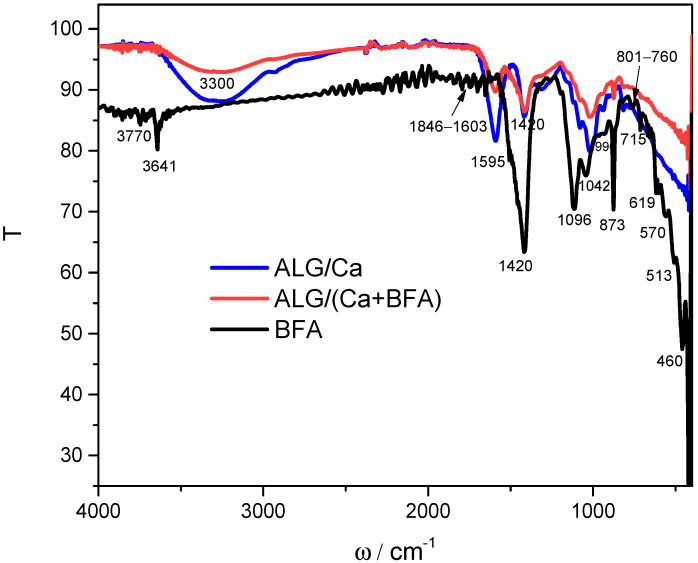
FTIR spectra of calcium alginate microspheres loaded with biomass fly ash and ALG/(Ca + BFA) (red line), calcium alginate microspheres and ALG/Ca (blue line), and biomass fly ash (BFA) (black line).

Information on molecular interactions between constituents in microsphere formulation was obtained by comparing the FTIR spectra of BFA, calcium alginate microspheres (ALG/Ca), and the microsphere formulation ALG/(Ca + BFA) (Figure 1). Biomass fly ash is a complex heterogeneous mixture of both amorphous and crystalline phases [12]. The BFA spectrum shows the most intensive absorption peaks corresponding to calcite (at 1420, 873, and 715 cm^−1^), quartz (at 1096 cm^−1^), and clay minerals (shoulder at 1042 cm^−1^) as well as at 3641 cm^−1^, attributed to OH stretching of the hydroxyl anions of portlandite in hydrated lime. A relatively low peak intensity indicates that the hydroxyl groups are unlikely to bond to each other via hydrogen bonds [20]. Peaks of lower intensity are attributed to quartz (in the ranges 1603–1846 cm^−1^ and 760–801 cm^−1^ as well as 460 and 513 cm^−1^) [21], arcanite (990, 619, and 570 cm^−1^) [22], periclases (at 3770 and 459 cm^−1^) [23], and various aluminosilicates (915 to 554 cm^−1^) [24]. A series of bands characterized by very low intensities can be seen in the middle. This indicates the presence of organic compounds in the traces left behind after coal combustion (e.g., bands associated with vibrations of C=O and/or C=C). However, in this case, an accurate explanation of the tape is very difficult. All of these bands are consistent with the literature data [25,26]. The BFA FTIR spectrum is consistent with the results of an X-ray analysis that showed that the main mineral phases of BFA are calcite (CaCO_3_), quartz (SiO_2_), lime (CaO), portlandite (Ca(OH)_2_), potassium oxide (K_2_O), periclase (MgO), and arcanite (K_2_SO_4_), with a smaller amount of other minerals such as aluminum/iron oxides [12].

The main characteristic bands of ALG/Ca correspond to hydroxyl stretching vibration around 3300 cm^−1^, peaks related to carboxylate groups (COO-) (asymmetric stretching vibrations at 1595 cm^−1^ and symmetric at 1420 cm^−1^), and the peak stretching vibrations of C-O-C groups at 1024 cm^−1^ [27]. Bands situated between 900 and 1200 cm^−1^ are characteristic of a polysaccharide structure.

Microspheres loaded with BFA showed the absence of characteristic BFA bands, confirming successful loading. Significant reductions in the peak intensities of all characteristic calcium alginate stretching vibrations (hydroxyl groups around 3300 cm^−1^, asymmetric COO- groups, and C-O-C groups) as well as small peak shifts attributed to asymmetric COO- groups (from 1595 to 1585 cm^−1^) and C-O-C groups assignable to mannuronic and guluronic acid (from 1024 to 975 cm^−1^) indicated a weakening of the hydrogen bonds and electrostatic interactions identified in ALG/Ca microspheres as dominant molecular interactions.

**Figure 2:**
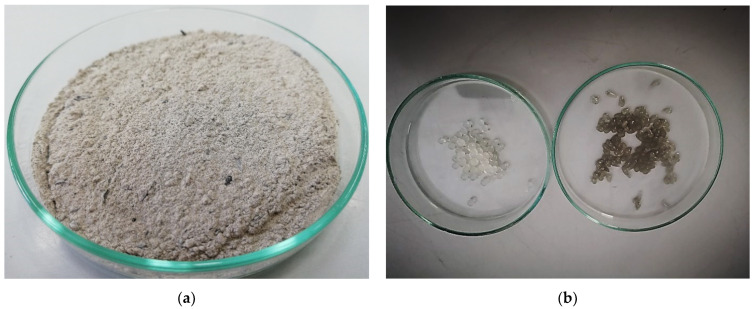
Photographs (light binocular) in a Petri dish (d = 9 cm) of (**a**) BFA and (**b**) microspheres without (ALG/CA) (left) and with BFA ALG/(Ca + BFA) (right).

The loading of BFA (Figure 2a) into microspheres (Figure 2b) caused a change in color from near milky white (ALG/Ca) to gray (ALG/(Ca + BFA)), but no significant change in the mean size (~3500 µm) was observed. The loss of water and humidity associated with biopolymer strain–relaxation processes during the drying process reduces the size of both microsphere types by approximately 50%. Drying to constant mass caused only a slight deformation of the spherical shape of the ALG/Ca microspheres, but those loaded with BFA became significantly irregular and wrinkled in appearance with visible BFA particles near the surface.

### 2.2. SEM-EDX Analysis

**Figure 3:**
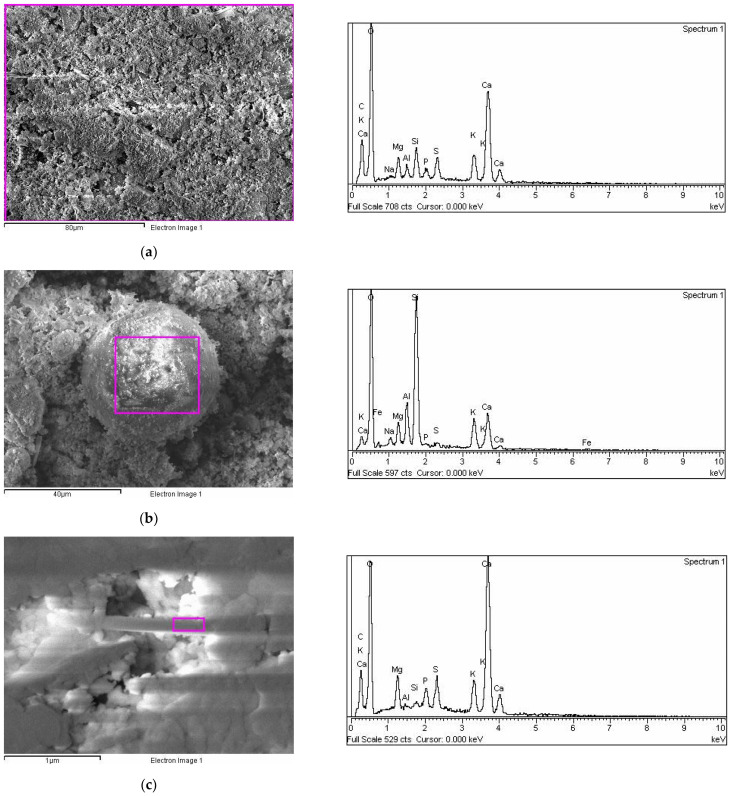
SEM microphotographs (bars are indicated) of (**a**) the whole, (**b**) spherical, and (**c**) needle-shaped BAF particles with surface elemental analysis using dispersive X-ray spectroscopy.

SEM images of crude BAF (Figure 3a) showed the presence of irregular along with some spherical (Figure 3b) and needle-shaped (Figure 3c) particles as well as irregular agglomerates. Figure 3a–c present differences in EDX analyses applied to the area nearest to the surface (the electron probe can penetrate to a depth of about 1 μm) of the whole sample, fragmented parts, and spherical and needle-shaped particles. An analysis of the whole sample showed the predominant presence of O, Ca, C, and K (Figure 3a). Other identified elements were Si, S, Mg, and less than 1% P, Al, Na. AAS spectrophotometry and flame photometry indicated that the identified elements were mostly in various oxides [12]. The crystalline appearance of a spherical particle with the highest Si percentage on the surface (Figure 3b) and the specific morphology (needle-shaped) with the highest percentage of Ca on the surface (Figure 3c) indicate that these particles seem to belong to quartz [28] and calcite [29], respectively. These assumptions confirmed the recently published microscopic/spectroscopic BFA characterization, which showed that the most abundant minerals are quartz and calcite [12].

**Figure 4:**
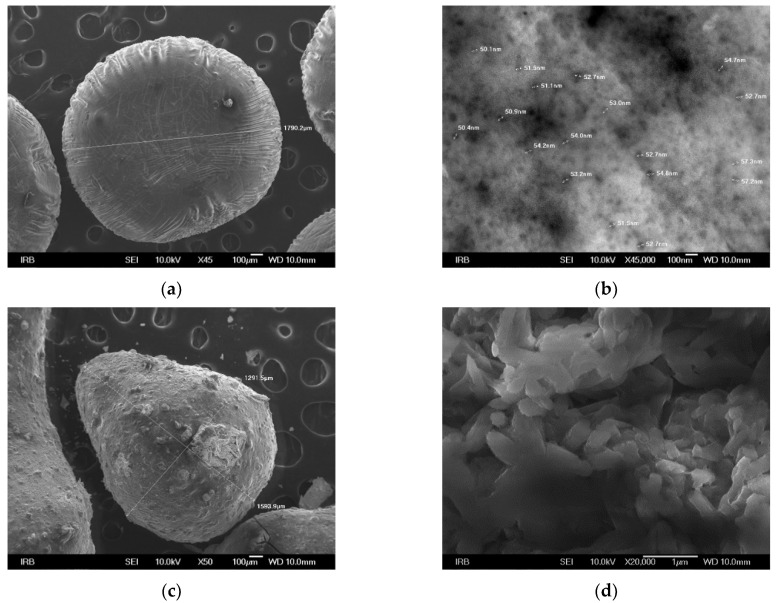
SEM microphotographs of calcium alginate microspheres (**a**,**b**) and microspheres loaded with BFA (**c**,**d**) at the various magnifications. Bars are denoted.

Dried ALG/Ca microspheres were spherical with an average diameter of ~1800 µm (Figure 4a). The enlarged image shows a highly porous surface with pore sizes from 0.54 to 0.160 µm (Figure 4b). After loading with BFA, microspheres became deformed and somewhat smaller (Figure 4c). The enlarged image exhibits many pores and irregular BFA particles localized near the surface (Figure 4d).

**Figure 5:**
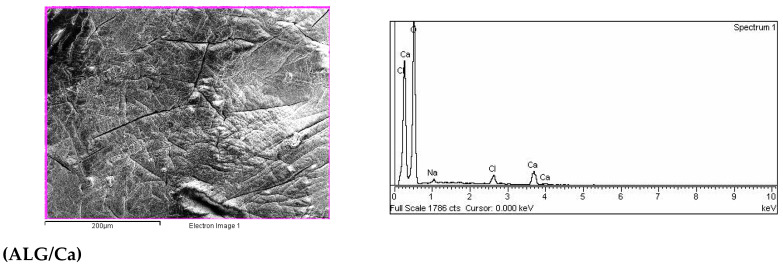

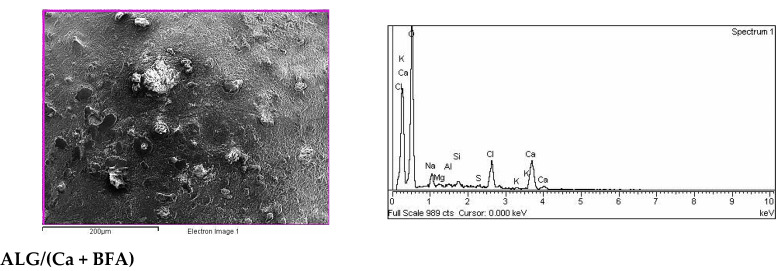
SEM microphotographs (bars are indicated) of ALG/Ca and ALG/(Ca + BAF) with surface elemental analysis using dispersive X-ray spectroscopy as denoted.

An EDS spectra analysis of the area nearest to the AG/Ca microsphere surface is shown in Figure 5, showing that the major elements are oxygen, carbon, and calcium. The small amounts of sodium and chloride detected were probably residues of compounds used during microsphere preparation. An elemental analysis of the ALG/(Ca + BAF) microsphere also showed the dominant presence of O, C, and Ca as well as the presence of Na, K, Cl, Mg, S, Si, and Al. The detection of BFA elements indicated that a part of the BFA localized near the surface.

### 2.3. AFM Analysis

**Figure 6:**
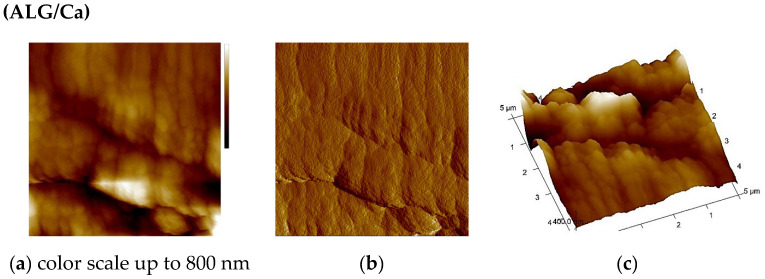

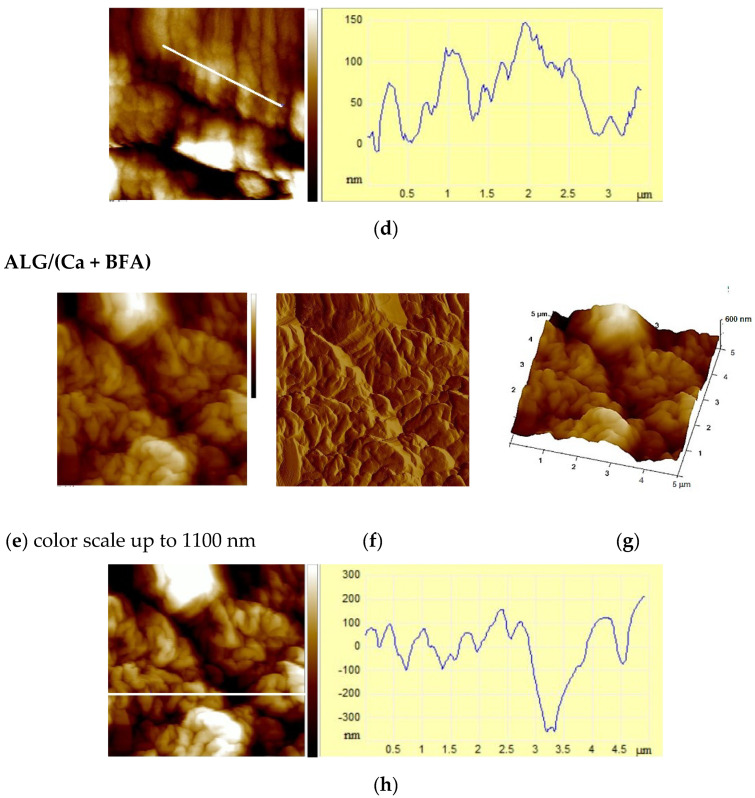
AFM images of ALG/Ca and ALG/(Ca + BFA) microspheres are shown as topographic (**a,e**) 2D height images of the scan area (5 × 5 μm^2^) as “top view”; (**b,f**) amplitude image of the microparticle surface in the scan area (5 × 5 μm^2^) (top view), (**c,g**) 3D topographic images of height data, and (**d,h**) section analysis profiles (right) along labeled white lines (left).

An AFM analysis of the ALG/Ca and ALG/(Ca + BFA) microspheres and those loaded with BFA was performed to complement the SEM surface morphology data (Figure 6). The scanned sample area represented by topographic images of the height data is shown as a “top view”, characterizing the morphology of each formulation, and as a “3D surface view” with a corresponding color scale, characterizing the 3D height topography of the formulation (Figure 6a–c). The characteristic vertical profile (“section analysis”) of individual microspheres, showing the quantitative 2D height analysis, is presented in Figure 6d. The surface of the ALG/Ca microspheres had a granular surface structure consisting of compactly stacked oval smooth granules in layers aligned mainly along one axis. The distribution of the lateral dimension of the grains was in the range of 300 nm to 500 nm, while the height of the grains above the microsphere surface was between 30 nm and 60 nm. Such a morphology was obtained during the preparation of ALG/Ca at a lower concentration of the gelling cation [27].

After the loading of BFA, the morphology took on completely different characteristics. The granules no longer retained their regular oval shape, and their surfaces were wrinkled. The overall lateral dimension of the granules, now elongated and distorted, had increased and ranged from 500 nm to 1500 nm, while the height of the granules above the microsphere surface was 50 to 200 nm. The roughness values also showed the effect of BFA on the roughness of the microsphere surface itself, which became hardened and less compact due to the incorporation of BFA (Table 1).

### 2.4. Swelling and In Vitro Release of K, Ca, and Mg from Microsphere Formulations

In addition to easier handling in the field or large areas, a very important property of microsphere formulation is the rate and mechanism of plant nutrient release. When dispersed in water, hydrophilic polymer microspheres swell by two basic molecular processes, the penetration of the water into microspheres and polymer stress relaxation (the transition of a glassy structure to a rubbery state) [30]. Swelling, like other physicochemical properties of alginate hydrogels, is highly dependent on crosslinking density and mainly determined by the calcium concentration, chemical composition (percentages of mannuronic acid and guluronic acid), and concentration of alginate [31]. The extent of the cooperative interactions of cations with the carboxylate groups of the guluronic acid residues of alginate determines the density of the crosslinked network influencing the properties of the gel. Using the swelling degree as a measure of the extent of crosslinking [32], the increase in swelling degree from 21% (ALG/Ca) to 25% (ALG/(Ca + BFA)) indicated the somewhat reduced density of the network structure and the increased cavity size inside the gel network, which accommodated water.

**Figure 7:**
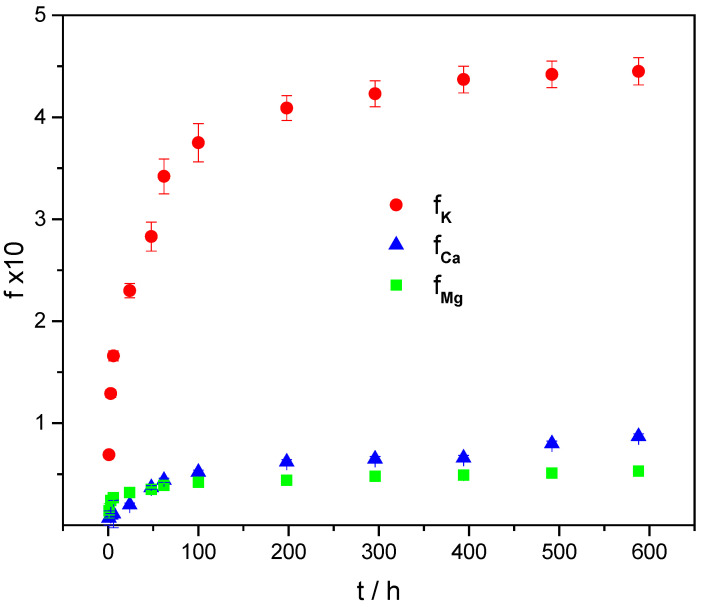
Released fraction of potassium (f_K_), calcium (f_Ca_), and magnesium (f_Mg_) cations from the microsphere formulation with time (t). The error bars indicate the standard deviations of the means.

After the dispersion, wetting, and swelling of ALG/(Ca + BFA) in distilled water, water-soluble BFA species were released from the microsphere formulations. Various physicochemical processes are involved in the release of active agents (swelling, diffusion through the network structure, dissolution in the medium, disintegration, dissolution or erosion of the structure, or their combination), but the most important rate-controlling release mechanisms from hydrophilic hydrogels are diffusion, swelling, and erosion [30]. The release experiments have been focused on the major BFA elements, which are important plant nutrients. The released K, Ca, and Mg concentrations are presented as the fraction of the cumulatively released amount with time (Figure 7). A set of release profiles shows differences in the amount and the release rates among the different nutrients. Potassium was released the most and the fastest, while the amounts and speeds of Ca and Mg were significantly lower.

The controlling mechanism and the release rates of potassium, calcium, and magnesium from the microsphere formulation were identified by analyzing the release profiles using the simple empirical Korsmeyer–Peppas model [33]:

(1) $$f={\mathrm{kt}}^{n}$$

where f represents the fraction of released ions, k is a kinetic constant characteristic of a particular system, incorporating the overall solute diffusion coefficient and geometric characteristics of a microsphere, and n is the release exponent, representing the release mechanism.

According to the model [33], the release exponent, n, can be characterized by three different release mechanisms. Values of n < 0.43 indicate the release is controlled by classical Fickian diffusion, n > 0.85 indicate the release is controlled by type II transport, involving polymer swelling and the relaxation of the polymeric matrix, whereas values of 0.43 > n > 0.85 show the anomalous transport kinetics determined by a combination of the two diffusion mechanisms and type II transport. Values of the release constant, k, and exponent, n, are listed in Table 2.

n values lower than 0.43 indicated that the release of measured cations was controlled by Fickian diffusion [33]. The highest release rate and the released amount of potassium compared to calcium or magnesium agreed with the higher concentration of leached potassium from wood ash granules or pellets [34,35].

**Figure 8:**
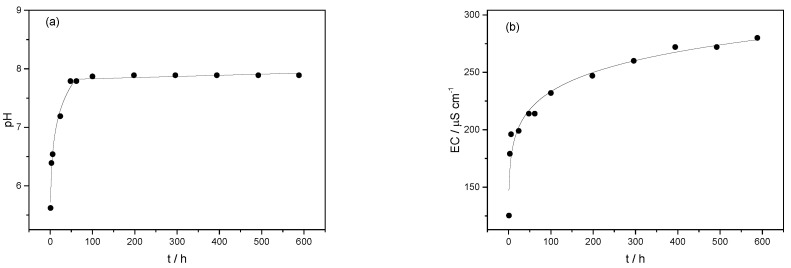
(**a**) The pH and (**b**) electrical conductivity (EC) variation with time (t) during the in vitro release from the microsphere formulation ALG/(Ca + BFA).

Changes in pH (Figure 8a) and electrical conductivity (EC) (Figure 8b) during the in vitro release studies followed release profiles. They were characterized by an initial rapid pH increase (up to 100 h) followed by slower release. In the first time interval, potassium was the main element released and contributed the most to the changes in pH and conductivity. Potassium reacts intensely with water, forming basic potassium hydroxide solutions, although the releasing and hydrolysis of other oxide constituents also contributed to the pH increase. The low alkalinity of the solution showed an additional advantage of applying encapsulated BFA compared to direct BFA application to soil, which causes a sudden increase in alkalinity. There was no change in the pH of the release solution in the second time interval due to two opposing processes: (i) the dissolution and hydrolysis of oxide constituents (such as K_2_O, CaO, etc.), which contributed to increasing the pH of the solution, and (ii) a slow dissolution of silica, resulting in the formation of silicic acid [36]. While the pH values remained almost constant in the second time interval, a continuous increase in EC was observed. This can be attributed to the slower release of all soluble anions and cations present in BFA [37].

The loading of calcium alginate microspheres with BFA alters the number of alginate strands held together in the three-dimensional network and thus changes the crosslinking density and the size of cavities inside the gel network, which accommodates water [38]. Calcium alginate microspheres are matrix (monolithic) devices with a controlled release of ingredients that diffuse through pores or channels in the gel phase [39]. In diffusion-controlled systems, K, Ca, and Mg diffuse, depending on their sizes and the space available between polymer chains [40]. To diffuse, cations must be dissolved, and the rates and amounts of K, Ca, and Mg released depend on the solubility of their minerals in a gel-like matrix and possible divalent cation exchange, mainly on mannuronic acid residues of alginate.

Kim et al. [41] have shown that the relative solubility of cations is consistent with the solubility of minerals in fly ash and belongs mostly to alkaline and alkaline-earth elements [13]. This means that the kinetics and amount of released cations from a microsphere formulation depend on the aqueous solubility of the species present in BFA as well as on the electrostatic interactions of divalent cations with alginate chains. Potassium is present in more soluble species compared to calcium or magnesium. A higher release rate compared to calcium or magnesium follows the observed higher potassium leaching rate from BFA pellets compared to Ca [13]. Differences in the release rate and amount released between calcium and magnesium cations stem from the fact that calcium species in BFA are relatively more soluble in water than magnesium species. Similarly, Du et al. [42] emphasized the importance of the role of nutrient solubility in the release from polymer-coated controlled-release compound fertilizer.

Based on the physicochemical properties and release mechanism, the prepared ALG/(Ca + BAF) microspheres have a great potential to be used for plant nutrition. Our future research will focus on greenhouse and outdoor plant applications to confirm BFA recycling in agriculture is an important part of the circular economy [43].

## 3. Materials and Methods

### 3.1. Materials

Alginic acid sodium salt (CAS number: 9005-38-3, M/G ratio of ∼1.56, molecular weight of 280,000 g mol^−1^) and calcium chloride dihydrate (CAS number: 10035-04-8) were purchased from Sigma Aldrich (Missouri, St.Louis, USA). All other chemicals were of analytical grade and were used as received without further purification.

Biomass fly ash material was obtained from the cogeneration biomass facility Viridas Biomass, Babina Greda, Croatia (max. net electrical power of 8.6 MW; max. heat power of 16 MW), located in the vicinity of the soil sampling area. The sampling and characterization of BFA used in this study were described extensively in a recent paper by Ondrašek et al. [12]. Used BFA does not contain heavy elements above the maximally permitted concentrations for soil conditioners and consequently could be applied as an inorganic soil conditioner for the amelioration of agricultural acidic soils.

#### Preparation of Microsphere Formulations

The microsphere formulation ALG/(Ca + BFA) was prepared by loading calcium alginate microspheres with BFA using a modified ionic gelation method [17,18]. ALG/(Ca + BFA) production by ionic gelation method involves dripping 500 mL of sodium alginate (2% *w*/*v*) with dispersed BFA (4 g) into 500 mL of calcium chloride dihydrate solution (1.5% *w*/*v*) using a syringe with a constant stirring. Before dripping, BFA was dispersed in the sodium alginate solution and homogenized for 1 h with Silverson Laboratory Mixers (Silverson Machines, Inc., London, UK). Microspheres without BFA (ALG/Ca) were prepared for comparison. The prepared microspheres were washed several times with distilled water, filtered through the filter funnel, and stored at 4 °C until further studies.

### 3.2. Methods

The Fourier transform infrared spectroscopy (FTIR) spectra were recorded with the IRTracer-100 Spectrophotometer (Shimadzu, Kyoto, Japan) with a QATR-10 attachment (Shimadzu, Kyoto, Japan) for measuring powder samples. The samples were powdered before measurement and scanned in the range of 400–4000 cm^−1^.

The optical imaging was performed using a BX60 optical microscope (Olympus Corp., Tokyo, Japan), and 100 microspheres were randomly selected from batches, produced in triplicate, to determine the size distribution by light binocular. The average diameters of wet and dry microparticles were determined using Olympus Soft Imaging Solutions GmbH, version E_LCmicro_09Okt2009.

The SEM analysis was performed with the use of a JSM-7000F field emission scanning electron microscope (Jeol Ltd., Tokyo, Japan) equipped with an EDS/INCA 350 energy-dispersive X-ray analyzer (Oxford Instruments Ltd., Abingdon, UK). The EDS standard by which the spectrometer was calibrated was annealed Ti foil (thickness 0.127 mm, Alfa Aesar, purity 99.99%+, CAS 7440-32-6), while the standards for measuring other elements were from the Oxford INCA database of the instrument itself. Dried samples for analysis were put on high-conductive graphite tape.

The atomic force microscopy (AFM) imaging was performed with a MultiMode 8-HR Scanning Probe Microscope with a Nanoscope IIIa Controller (Bruker, Billerica, MA, USA) with SJV-JV-130 V (“J” vertical engagement scanner(JV), 125 μm, Bruker Instruments, Inc.) and tapping mode silicon tips (R-TESPA, Bruker, nom. freq. 300 kHz, nom. spring constant of 40 N/m). In this way, three-dimensional information about the surface topology was obtained and the roughness was quantified. The samples were rinsed three times with 50 μL of MiliQ water to remove all remaining contaminants and deposited as microspheres on the mica substrate. All AFM images were performed on three different regions of each sample to ensure consistency in the obtained results.

#### Swelling Degree and Release of Potassium, Calcium, and Magnesium from Microsphere Formulation

A detailed procedure for the determination of the swelling degree (S_w_) was previously described [44]. S_w_ was calculated using the equation:

(2) $$S_{w}=\frac{w_{t}-w_{0}}{w_{0}}\;\times \;100\;$$

where w_t_ is the weight of the swollen microspheres and w_0_ is their initial weight.

In vitro release studies of microspheres were carried out at room temperature in the system formed by putting 4 g of microspheres in 100 mL of distilled water, as previously described [45]. The concentrations of released K, Ca, and Mg were determined by atomic absorption spectrometry (ASS) (HRN ISO 11466:2004). The results are presented as the fraction of potassium (f_K_), calcium (f_Ca_), or magnesium (f_Mg_) using the equation:

(3) $$f=\frac{R_{t}}{R_{\mathrm{tot}}}$$

where f represents the fraction of cumulatively released calcium, potassium, or magnesium, R_t_ is the calcium, potassium, or magnesium released at time t, and R_tot_ is the total amount of calcium, potassium, or magnesium loaded in the microsphere formulation. The concentration of released cations was determined by an atomic absorption spectrometer (Solar, Thermo Scientific, Abingdon, UK).

During release, the pH and conductivity changes were measured by the Mettler Toledo pH/Conductivity meter (Zagreb, Croatia). The results were statistically analyzed with Microsoft Excel 2016 and the XLSTAT statistical software add-on. The data are shown as mean values ± standard deviations.

## 4. Conclusions

A new soil amendment based on biomass fly ash encapsulated in calcium alginate microspheres was prepared and characterized. BFA loading weakened hydrogen bonding and electrostatic interactions in the microsphere formulation, thereby altering the crosslinking density of the three-dimensional network structure of calcium alginate and thus altering the surface morphology and topology.

Diffusion was detected as a mechanism controlling K, Ca, and Mg release, but the kinetics and released amounts from ALG/(Ca + BFA) differed significantly. The amounts of Ca and Mg released were smaller, and the release rates were slower compared to K due to the differences in the solubility of their minerals in a gel-like matrix as well as in the interactions of divalent cations with alginate chains

The physicochemical characteristics and nutrient release profiles revealed that the encapsulation of BFA in calcium alginate microspheres has great potential for application in agriculture. Encapsulation may provide a better method of BFA transportation, with reduced storage costs and easier handling in the field or on large surfaces. The benefits are multiple, from reduced need for BFA landfills to the safe return of nutrients to the environment, the reduced use of agrochemicals, and the prolonged delivery of nutrients to plants.

## Figures and Tables

**Table 1:** Roughness parameters, average roughness (*R*_a_), root mean square of roughness (*R*_ms_), and *Z* range for the ALG/Ca and ALG/(Ca + BFA) microspheres.

Sample	*R*_a_/nm	*R*_ms_/nm	*Z*/nm
ALG/Ca	76 ± 1	106 ± 2	1127 ± 21
ALG/(Ca + BFA)	122 ± 2	159 ± 1	1054 ± 21

**Table 2:** Values of the release constant (k/h), exponent (n), and correlation coefficient (R^2^) of potassium, calcium, and magnesium released from the microsphere formulation ALG/(Ca + BFA).

ALG/(Ca + BFA)	k	n	R^2^
K	0.12	0.22	0.98
Ca	0.019	0.16	0.98
Mg	0.009	0.36	0.97

## Abbreviations

BFA: biomass fly ash; ALG, alginate; ALG/Ca, calcium alginate microsphere; ALG/(Ca + BFA), microsphere formulation; FTIR, Fourier transform infrared spectroscopy; LB, light binocular, OM, optical microscopy; SEM, scanning electron microscopy; AFM, atomic force microscopy.
